# Regulation of Apoptosis by Inhibitors of Apoptosis (IAPs)

**DOI:** 10.3390/cells2010163

**Published:** 2013-03-14

**Authors:** Jean Berthelet, Laurence Dubrez

**Affiliations:** 1 Institut National de la Santé et de la Recherche Médicale (Inserm) UMR866 Dijon F-21079 France; E-Mail: berthelet.jean@gmail.com; 2 Université de Bourgogne, Dijon F-21079, France

**Keywords:** KeywordsDIAP1, XIAP, cIAPs, apoptosis, caspases, RIP, apoptosome, IAP antagonists

## Abstract

Abstract Inhibitors of Apoptosis (IAPs) are a family of proteins with various biological functions including regulation of innate immunity and inflammation, cell proliferation, cell migration and apoptosis. They are characterized by the presence of at least one *N*-terminal baculoviral IAP repeat (BIR) domain involved in protein-protein interaction. Most of them also contain a *C*-terminal RING domain conferring an E3-ubiquitin ligase activity. In drosophila, IAPs are essential to ensure cell survival, preventing the uncontrolled activation of the apoptotic protease caspases. In mammals, IAPs can also regulate apoptosis through controlling caspase activity and caspase-activating platform formation. Mammalian IAPs, mainly X-linked IAP (XIAP) and cellular IAPs (cIAPs) appeared to be important determinants of the response of cells to endogenous or exogenous cellular injuries, able to convert the survival signal into a cell death-inducing signal. This review highlights the role of IAP in regulating apoptosis in Drosophila and Mammals.

## 1. Introduction

Apoptosis is a highly evolutionary conserved mechanism of cell death triggered by a large range of extracellular or intracellular stimuli including developmental signals, environmental and intracellular stress. This is a genetically controlled process, playing a major role in normal development and tissue homeostasis. It is considered as a potent mechanism of tumour protection, ensuring the selective removal of supernumerary, undesirable or damaged cells. It is also essential in immune response and constitutes an efficient strategy of antiviral defence [1]. Viruses have developed strategies in order to overcome this protection mechanism, allowing the replication and the spreading of virus. A genetic screen aiming to identify viral proteins that evade virus-induced cell death in host cells revealed Inhibitors of Apoptosis (IAPs) as potent inhibitors of apoptosis in insect cells [2]. IAP homologs were then identified in yeasts, nematode, insects, fishes and mammals, sharing structural features [3]. Only a subset of IAPs functions as apoptosis regulators, many of them display non-apoptotic functions [4,5]. For example, Drosophila IAP2 (DIAP2) and mammalian cellular IAPs (cIAPs) are important intermediates in Tumour Necrosis Factor (TNF) Receptor (TNFR) superfamily and Nuclear Factor-kappaB (NF-κB) activating signalling pathways (for review, see [6,7,8]), regulating cell differentiation [9], cell motility [10], pro-inflammatory and immune response [5]; cIAP1 regulates cell proliferation through its capacity to control the activity of transcription factors such as c-myc [11] and E2F1 [12]; survivin regulates cell division by controlling cytodieresis [13]. This review focuses on the role of IAPs in the regulation of apoptosis, in insect and mammalian cells.

## 2. Structural Features of IAPs (Figure 1)

**Figure 1:**
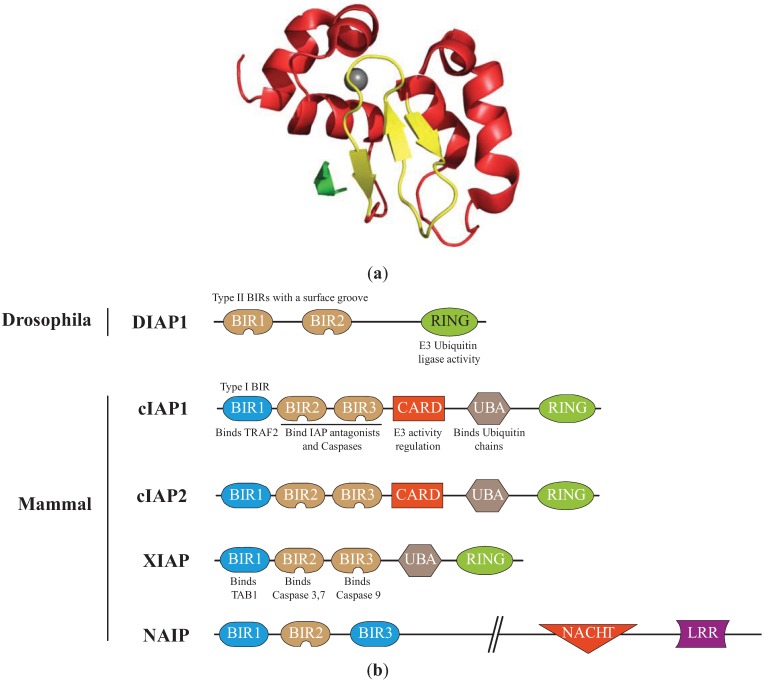
Structure of the Inhibitors of apoptosis. (**a**) Structure of the cellular IAP1 (cIAP1)-baculoviral IAP repeat (BIR)3 bound to the caspase-9 *N*-terminal peptide [30]. BIR3 is organized in four α-helices (red) and 3 β-strand sheets (yellow) maintained by zinc ion (grey). The interaction involved the surface hydrophobic groove of cIAP1 and the *N*-terminal peptide (ATPFQ) of the caspase 9 sub-unit (Constructed using the PyMOL Molecular Graphics System). (**b**)Representation of IAPs involved in the regulation of apoptosis. The type I baculoviral IAP repeats (BIR, blue) of cIAPs and X-linked IAP (XIAP) can bind to cell signalling intermediates TNFR associated factor 2 (TRAF2) and Transforming Growth Factor beta-activated kinase 1-binding protein 1 (TAB1), respectively. The type II BIRs (brown) contain a surface hydrophobic groove allowing the interaction with IBM found in caspase sub-units and IAP antagonists. The ubiquitin Associated (UBA) domain binds ubiquitin chains. The caspase recruitment domain (CARD) is a module of regulation of the RING E3-ubiquitin ligase activity. The RING domain confers to IAPs an E3-ubiquitin ligase activity. NACHT (domain present in NAIP, CIITA, HETE and TP1). LRR: Leucine Rich Repeat.

The IAP family is defined by the presence of one to three tandem specific motifs of about 70 residues named Baculovirus Iap Repeat (BIR) located at the *N*-terminal end of the protein [14] (Figure 1). The core component of the BIRs is a consensus Cys/His motif (GX_2_YX_4_DX_3_CX_2_CX_6_WX_9_HX_6–10_C) that coordinates a single zinc ion [14,15]. It is organized in series of short α-helices with intervening β-sheets, forming a specific folded structure stabilized by the Zinc (Figure 1). BIRs are protein interacting modules with specific distinct binding properties [16]. Most of them (referred to as type II BIR) form a surface hydrophobic groove that binds a conserved tetrapeptide motif called IAP Binding Motifs (IBMs, A(K, T, V, I)(P, A, E)(F, E, I, S, Y)). IBMs were found in the extreme *N*-terminus of sub-units of some caspases and in IAP antagonists [17,18]. The *N*-terminal exposure of IBM is required for the recognition and binding by IAPs. Thus, BIR hydrophobic groove can only bind processed, activated caspases. Type II BIRs are essential for the anti-apoptotic properties of IAPs. Type I BIRs do not bind IBM but can interact by distinct mode to another set of proteins mainly involved in cell signalling pathways [19,20,21]. The second conserved motif important for the anti-apoptotic activity of IAPs is the *C*-terminal RING (really interesting new gene) zinc-finger that displays an E3-ubiquitin ligase activity. Thanks to this domain, IAPs can catalyse the covalent conjugation, to a lysine of target partner proteins, of NEDD8 (neural precursor cell expressed developmentally downregulated protein 8) [22,23] or a single ubiquitin (Ub) molecule (monoubiquination) or polyubiquitin chains formed by the binding of the *C*-terminal Glycine of Ub to the Lysine (mainly K11, K48 or K63) residue of another. K48-linked chains usually target the protein for 26S proteasomal degradation, while mono-ubiquitin or K63 or K11-linked ubiquitin chains modulate the activity or the cellular distribution of target proteins. IAPs can also mediate their own ubiquitination [24]. Furthermore, the RING enables homo- or heterodimerization of IAPs that regulates their stability and possibly their activity [24,25,26]. In addition to the BIRs and RING, other conserved protein domains can be found in IAPs including a central caspase recruitment domain (CARD) that regulates E3-ubiquitin ligase activity [27], a Ub associated (UBA) domain that can recognize mono- and poly-Ub chains and that allows the recruitment of IAP in protein complexes (Figure 1) [28,29].

## 3. Apoptotic Signalling Pathways

Apoptosis is a highly evolutionary conserved process. Both vertebrate and invertebrate apoptotic pathways are mediated by a sequential activation of cysteine proteases from caspase family which are responsible for characteristic morphological and biochemical changes [31]. Caspases are expressed as inactive-zymogens consisting of one prodomain and two active sub-units (a small and a large one). They are sub-divided into initiator and effector caspases, depending of the length of the pro-domain and the mechanism of activation. The initiator caspases possess a long pro-domain allowing their recruitment by adaptors into caspase-activating complexes. These molecular platforms provide proximity required for caspase homodimerisation and self-activation. They activate the effector caspases by a proteolytic cleavage allowing the assembly of two small and two large sub-units into an active tetramer. Effector caspases cleave broad spectrum of cellular proteins leading to cell dismantlement. Members of Caspase family are also involved in non-apoptotic processes such as inflammatory response, cell proliferation and differentiation [31].

## 4. Regulation of Apoptosis by IAPs in Invertebrates

Insect models such *drosophila melanogaster* and *spodoptera frugiperda* provide powerful genetic tools to dissect cell death pathways. Apoptosis is essential to eliminate damaged and unwanted cells, but it also controls organ morphogenesis during drosophila embryogenesis [32]. A genetic screen in cell death defective drosophila embryos identified IAP genes that regulate developmental programmed cell death [33,34,35,36,37].

### 4.1. Drosophila Apoptotic Signalling Pathway

**Figure 2:**
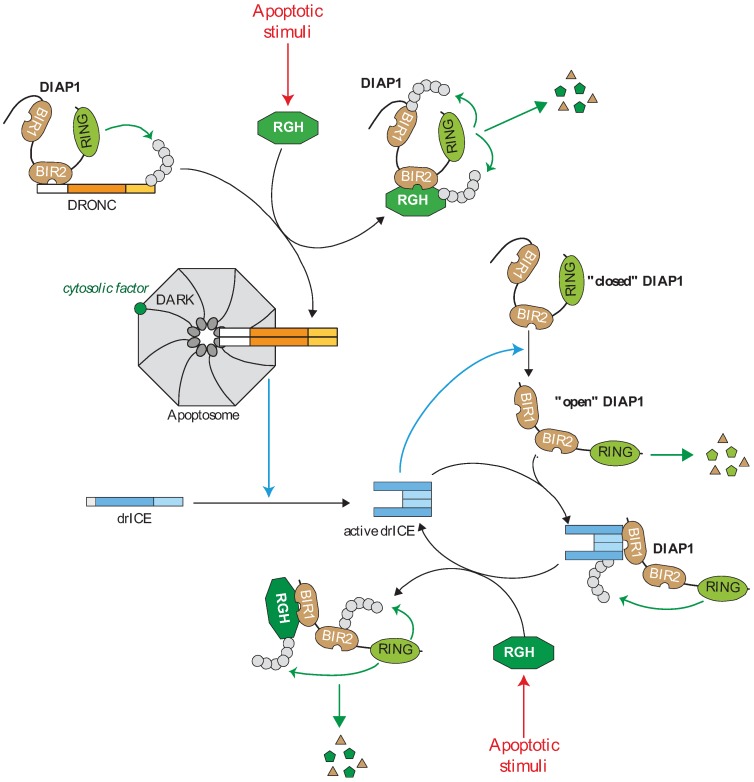
Regulation of the caspase cascade by IAPs in drosophila. In living cells, the caspase activating cascade is maintained in check by a direct interaction of caspases with the Drosophila IAP1 (DIAP1). The DIAP1 BIR2 binds to the prodomain of the apoptotic initiator DROsophila Nedd-2-like Caspase (DRONC) and the RING induces DRONC ubiquitination preventing apoptosome assembly. DIAP1 is expressed in “closed conformation” in which the *N*-terminal sequence hides BIR1 surface groove. Effector caspase mediates the cleavage of the *N*-extremity of DIAP1 that releases the BIR1 domain which in turn interacts with the IBM exposed on the active form of effector drosophila melanogaster Interleukin-1-converting enzyme/Ced-3 related protease (drICE). DIAP1 inhibits drICE activity through a degradative or non-degradative ubiquitination or neddylation. The “open” form of DIAP1 is highly unstable and rapidly degraded by the *N*-end-rule-associated degradation machinery. Apoptotic stimuli induce the expression of the IBM-carrying IAP antagonists Reaper, Grim or Hid (RGH) wich strongly bind and neutralize DIAP1 through IBM-BIRs interaction. The IAP antagonist-DIAP1 interaction promotes DIAP1-autoubiquitination and degradation.

In drosophila, the apoptotic initiator caspase DRONC (DROsophila Nedd-2-like Caspase) is involved in almost all forms of apoptosis [38,39,40,41,42,43]. It is activated by dimerization through the recruitment by the Apaf-1 (apoptotic protease activating factor 1) ortholog DARK (Drosophila Apaf-1 related killer) at the caspase-activating platform apoptosome [40,41,42,44,45]. Unlike mammalian models, cytosolic cytochrome c seems dispensable for the *in vitro* apoptosome assembly [45,46,47], although the requirement for a cytosolic factor has been demonstrated [48]. Once activated, DRONC activates the effector caspase drICE (drosophila melanogaster Interleukin-1-converting enzyme/Ced-3 related protease) and DCP-1 (death caspase-1) [44,49,50] (Figure 2). Caspases and DARK are constitutively expressed. In the absence of apoptotic inducers, the cell death machinery is frozen by the presence of important regulatory mechanisms. Among them, IAPs prevent unexpected assembly of apoptosome and caspase cascade activation [3] (Figure 2).

### 4.2. Drosophila IAPs as Caspase Inhibitors

The drosophila genome encodes at least four members of IAP family: drosophila IAP1 (DIAP1), drosophila IAP2 (DIAP2), DETERIN and drosophila BIR repeat-containing ubiquitin-conjugating (dBRUCE) [3]. DIAP1 (Figure 1), referred as a “gatekeeper of death” [3], is essential to ensure cell survival, neutralization of DIAP1 being necessary and sufficient to trigger apoptosis [33,40,44]. Loss-of-function mutations in DIAP1encoding gene (*thread*) lead to early embryonic death with a massive caspase-dependent apoptosis [33,51,52]. *Diap1*-deficiency-induced cell death is rescued by the inactivation of DRONC or DARK [40]. Moreover, DIAP1 inhibits apoptosis induced by the expression of either the initiator DRONC or the executor drICE caspase [38,39,53], suggesting that DIAP1 can control canonical apoptotic pathway at different steps (Figure 2).

DIAP1 contains two tandem BIRs and one RING domain providing an E3-ubiquitin ligase activity (Figure 1b). It can physically interact with both DRONC and drICE and, thanks to the RING, induces their ubiquitination [38,39,41,53,54,55]. The BIR2 DIAP1 binds a 12-residue linker region located in the prodomain of DRONC [38,39,41,55,56]. Although essential, binding is not sufficient for DRONC regulation *in vivo* since *diap1* mutant able to bind DRONC but lacking E3-ubiquitin ligase activity are inefficient to prevent apoptosis [54]. The consequence of DIAP1-mediated DRONC ubiquitination is still unclear. It has been suggested that ubiquitination leads to proteasome-mediated depletion of DRONC, preventing its accumulation in living cells [44,57]. However, a more recent report demonstrated that DIAP1-mediated ubiquitination of full length DRONC inhibits its activation and processing through a non-degradative mechanism [58]. The level of activation of DRONC is correlated with the amount of active apoptosome formed by DRONC and the adaptor DARK. A feedback regulation of the expression of both apoptosome components has been described [57]. The adaptor DARK can decrease the level of DRONC protein expression and conversely, DRONC lowers DARK protein level by a proteolytic cleavage. The ubiquitin ligase activity of DIAP1 is required for this process, suggesting that DIAP1 also regulates apoptosome assembly [57].

Unlike DRONC, only the active forms of effector caspases bind DIAP1 [53,56]. The mechanisms of binding have been extensively investigated and involve the surface groove of DIAP1 BIR1 domain that specifically recognizes the IBM found on the *N*-terminus of the large sub-unit of executive caspases, which becomes exposed after activating proteolytic cleavage [39,53,56,59]. In native form, DIAP1 stays in a closed conformation [60,61] in which its *N*-terminal sequence binds and occupies the BIR1 surface groove, preventing DIAP1-drICE interaction [61]. Auto-inhibition of DIAP1 is disabled by drICE caspase-mediated cleavage of DIAP1 after Asp20, that releases the BIR1 surface groove and renders fully competent DIAP1 to bind and inhibit active drICE [60,61,62,63]. Such feedback inhibition could allow regulation of the level of caspase activation in apoptotic or non-apoptotic process. Several mechanisms have been proposed to explain the DIAP1-mediated caspase inhibition including degradative and non-degradative ubiquitination [64] and neddylation [22]. The caspase-mediated cleavage at position 20 renders DIAPs highly unstable, exposing an Asn at *N*-terminal position, which is an acceptor site for the *N*-end-rule-associated degradation machinery [60,63,65]. 

Thus, drosophila cell survival is ensured by a highly regulated control of the stability of DIAP1 and associated caspases. Although DIAP2 and dBRUCE are also able to bind and control the activity of caspases, their role in apoptosis seem much more limited. A *diap2* mutation mainly affects innate immunity because of the capacity of DIAP2 to control the non-apoptotic caspase DREDD *(*Death related ced-3/Nedd2-like protein) [66,67] and *dBruce* mutation causes male sterility because of its ability to regulate the caspases required for spermatogenesis process [68].

### 4.3. Drosophila IAP Antagonists from Reaper Family

Drosophila apoptosis requires the neutralization or destruction of DIAP1, allowing the DARK-mediated DRONC activation. A genetic analysis of defective mutant for developmental cell death revealed the requirement of *reaper* (*rpr*), *head involution defective* (*hid*) and *grim* in apoptosis induction [33,34,35,36,37]. These proteins share a *N*-terminal IBM recognized by the IBM groove of DIAP1 BIR1 and BIR2, and prevent DIAP1 to bind and neutralize caspases [33,37,51,55,69,70,71]. Along with Sickle and Jafrac2, two other IBM-containing proteins [72,73,74,75], they are referred as IAP antagonists. IAP antagonist-DIAP1 interaction also promotes DIAP1-auto-ubiquitination and degradation [71,74,76,77,78]. Overexpression of IAP antagonists can induce apoptosis in several insect and non-insect cells. IAP antagonists cooperate to connect cell death stimuli with apoptotic signalling pathways. Reaper, Grim, Hid and Sickle are transcriptionally up-regulated in response to different apoptotic stimuli including developmental signals and DNA damages [73], and Jafrac2 is activated by apoptotic stimuli induced-removal of the *N*-terminal endoplasmic reticulum (ER) signal peptide that leads to the exposition of IBM [72].

### 4.4. Viral Anti-Apoptotic IAPs

Insect viruses including baculoviruses, iridoviruses, entomopoxviruses encode members of IAPs with anti-apoptotic properties, preventing death of infected cells and promoting virus multiplication [2,79,80]. Viral IAPs inhibit the activation of initiator caspases but seem inefficient to block downstream effector caspases [81]. The analysis of binding specificity of viral IAPs suggested that they inhibit apoptosis through their capacity to bind and neutralize IBM-containing IAP antagonists through an IBM-BIR interaction [36,80,82].

## 5. Regulation of Apoptosis by IAP in Mammals

### 5.1. Mammalian IAPs

The human genome encodes 8 members of IAP family involved in innate immune response, cell division, cell proliferation and cell death pathways [6]. Among human IAPs, X-linked IAP (XIAP) and cellular IAP1 and 2 (cIAP1/2) share several properties with DIAP1 including the capacity to bind caspases and IAP antagonists through the BIR, and the ability of self ubiquitination and ubiquitination and Neddylation of caspases via the RING domain. cIAP1, cIAP2 and XIAP own three tandem BIR domains, one UBA and one *C*-term RING domain (Figure 1). In addition, cIAPs contain a central CARD domain. The BIR1 is a type I BIR (unable to bind IBM) required for cell signalling activity. The XIAP BIR1 binds the adaptor TAB1 (Transforming Growth Factor beta-activated kinase 1 (TAK1)-binding protein 1) [19]. TAB1 is involved in transforming growth factor β (TGF-β) and bone morphogenetic protein (BMP) signalling pathways, and binds the kinase TAK1 (TGF-β-activated kinase 1) controlling NF-κB and MAP (mitogen-activated protein kinase) activating signaling pathways. The cIAP BIR1 binds the adaptor TRAF2 (TNFR associated factor 2) which bridges receptors from TNFR superfamily to downstream signalling pathways [20,25,83]. The BIR2 and BIR3 are type II BIRs able to bind IBM motifs exposed in active tetrameric Caspases and IAP antagonists [17,84,85]. The deletion or down-regulation of cIAPs or XIAP does not usually trigger apoptotis but sensitizes cells to extracellular or intracellular apoptotic inducers. XIAP and cIAPs demonstrate overlapping activities, which render difficult their functional analysis. Knocking out (KO) of single *iap* gene in mouse does not lead to obvious developmental abnormalities [86,87], however, a combined deletion of *ciap1* with *ciap2* or *xiap* in mice resulted in mid-embryonic lethality due to cardiovascular failure [88]. The main activity of cIAP1 and cIAP2 likely involves their ability to regulate the NF-κB activating signalling pathway in innate immune responses (reviewed by [6]). Although XIAP also displays some signalling activities in TGF-β/BMP and NF-κB signalling pathways [19], it is considered as the most potent mammalian IAP apoptotic regulator, able to directly inhibit caspase activity [84].

### 5.2. Mammalian Apoptotic Signalling Pathways

**Figure 3:**
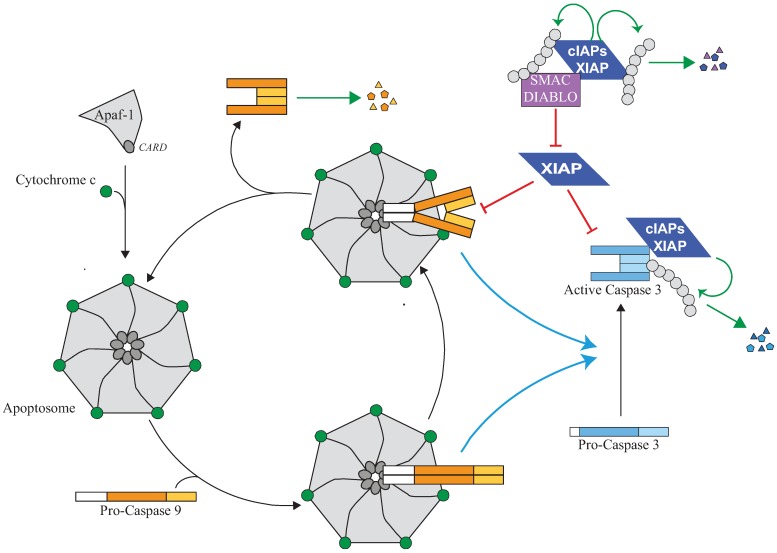
Regulation of the apoptosome and caspase activity by IAPs. The release of cytochrome-c from mitochondria which occurs during intrinsic pathway of apoptosis triggers an ATP-dependent conformational change and oligomerisation of the adaptor apoptotic protease activating factor 1 (Apaf-1) in a heptameric complex apoptosome. Apaf-1 then recruits Caspase-9 via its pro-domain through a homotypic CARD-CARD interaction. Caspase-9 is activated by homodimerisation and promotes the activating cleavage of effector caspase-3 leading to apoptosis. Caspase-9 undergoes autocatalytic processing and is then quickly disconnected from the apoptosome which is free to recruit a new pro-caspase-9. XIAP can control caspase activating pathway at several steps. First, XIAP is present in the apoptosome where it directly binds processed caspase-9 and inhibits its activity. The inhibition of caspase-9 by XIAP could stabilize the caspase-9 apoptosome complex and block the cycle of caspase-9 activation. Second, XIAP can directly bind and inhibit active effector caspase-3. XIAP can inhibit caspase activity by hindering substrate accessibility or hiding the protease catalytic residue, and/or by promoting ubiquitination or neddylation. Although unable to inhibit their activity, cIAP1 can bind to processed caspases and promote their ubiquitination. An IBM-dependent binding of IAP antagonists such as Smac/Diablo prevents XIAP-mediated caspase inhibition while cIAPs could interfere with neutralizing binding XIAP-Smac/Diablo.

Mammalian cells contain four apoptotic initiator caspases (caspase-2, -8, -9 and -10) solicited by different stimuli. The closest DRONC homolog is caspase-9 involved in a mitochondria-dependent apoptotic pathway, so-called intrinsic pathway [89,90]. It is activated in response to a large range of intracellular or extracellular stimuli which trigger a Bcl-2 (B-cell lymphoma-2) family member-dependent mitochondrial outer membrane permeabilization, resulting in the release of pro-apoptotic molecules including cytochrome-c and the IAP antagonists Smac/Diablo (second mitochondria-derived activator of caspases/direct IAP-binding protein with low pI) and Omi/HtrA2 (Omi stress-regulated endoprotease/High temperature requirement protein A2) [91,92]. Once cytoplasmic, cytochrome-c triggers the oligomerization of the adaptor Apaf-1 (Apoptotic peptidase activating factor 1) which recruits pro-caspase-9 allowing its activation at the apoptosome (Figure 3) [89]. Caspase-8 and -10 are activated in response to the engagement of death receptor from TNFR superfamily. Stimulation of Fas (DR2, CD95) or Trail (TNF-related apoptosis-inducing ligand) Receptor I or II (DR4 and DR5) induces the recruitment of the adaptor FADD (Fas-associated death domain protein), which then recruits and activates pro-caspase-8 or -10 in a receptor-associated platform named DISC (death-inducing signalling complex) [90]. FADD can also induced caspase-8 and -10 activation in cytoplasmic platforms such as Complexes-II or Ripoptosome [93,94,95]. TNFR1 stimulation induces the assembly of membrane associated oligomeric complex which transduces survival or pro-inflammatory signal. When survival pathways are blocked, a secondary cytoplasmic caspase-activating complex named Complex-II is formed, composed, in addition to the adaptor and the caspase, of the adaptor TRADD (TNFR1-associated Death domain) or the kinase RIP1 (Receptor interacting protein 1) [90]. Ripoptosome also contains the kinase RIP1 and is assembled in response to genotoxic stress, Tweak engagement or Toll-like receptor 3 stimulation [94,95]. Caspase-2 is recruited and activated in response to DNA damages by the adaptor RAIDD (receptor-interacting protein-associated ICH-1/CED-3 homologous protein with a DD) in a soluble platform named PIDDosome that contains the protein PIDD (p53-induced protein with death domain) [90]. Caspase-2 can also be activated in response of ER stress or bacterial toxin but the exact mechanisms of its activation is not well established [96,97].

All of these apoptotic pathways converge to the proteolytic activation of effector caspase-3 and -7.

### 5.3. Regulation of Apoptosome and Caspase-9 Activity by IAPs

The core component of Apoptosome is the adaptor Apaf-1. Binding of cytochrome-c triggers an ATP-dependent conformational change and oligomerisation of Apaf-1 in a heptameric complex in which the CARD domain of Apaf-1 forms a central ring structure that recruits pro-caspase-9 through the CARD found in its prodomain (Figure 3) (reviewed in [89]). This provides proximity required for oligomerization and self-activation of caspase-9. Once activated, caspase-9 undergoes autocatalytic processing [98] and activates effector caspases. It is then quickly disconnected from the apoptosome and inactivated, and possibly replaced in the apoptosome by a new pro-caspase-9 [98] (Figure 3). The presence of caspase inhibitors significantly reduces the dissociation rate of caspase-9 from the apoptosome blocking the cycle of activation of caspase [99]. Thus, caspase-9 activation is a dynamic, highly regulated process. XIAP likely takes part to these mechanisms of regulation of apoptosome activity (Figure 3). It is normally present in the apoptosome complex [17,100]. It does not influence pro-caspase-9 auto-processing but inhibits the activity of processed caspase-9 and then the activation of effector caspases [17,100]. It can directly bind and inhibit the activity of processed-caspases-9 by a two-site binding mechanism [84]. First, the surface groove of BIR3 binds the IBM, exposed at the *N-*terminus of the active small sub-unit after caspase-9 processing [17]. Second, the *C*-terminal extremity of BIR3 binds the dimer interface of caspase-9, preventing caspase-9 dimerization and hiding the catalytic residue [17,101]. Thus, XIAP could control apoptosome by inhibiting the activity of caspase-9 and by interfering with the cycle of activation of caspase-9 (Figure 3). Interestingly, a feedback regulation of apoptosome by caspase-3, which amplifies apoptotic process, has been described. Caspase-9 contains two caspase-3 cleavage sites. The first one produces the two active sub-units, and the second removes the IBM of the small active sub-unit, and then release caspase-9 from XIAP regulation [102]. Apoptosome activity is also regulated by the amount of cytochrome-c, Apaf-1 and pro-caspase-9 available [89]. Interestingly, the capacity of XIAP to control capase-9 activity appears to be directly correlated with the level of Apaf-1 and apoptosome activity. For example, XIAP effectively regulates sensitivity to apoptotic stimuli in cells expressing a low level of Apaf-1 such as terminally differentiated neuronal cells and cardiomyocytes [103,104].

Among mammalian IAPs, NAIP has also been detected in the apoptosome [105]. NAIP is an atypical IAP since it owns, in addition to the BIRs, a central NACHT (domain present in NAIP, CIITA, HET E and TP1) and a *C*-terminus LRR (Leucine Rich repeat) region [106] (Figure 1). Both domains are characteristic of NLR (NOD (nucleotide binding and oligomerization domain)-like receptor) family involved in intracellular recognition of microbial production and in the formation of the inflammatory caspase activation complex [107]. In contrast to other IAPs, NAIP can interact with pro-caspase-9. It inhibits its autocatalytic processing and the activation of effector caspase. This interaction involves the BIR3 domain of NAIP and is IBM-independent [105,108].

### 5.4. Regulation of Effector Caspases by IAPs

XIAP can also directly bind and inhibit effector caspases-3 and -7 (Figure 3) [109,110,111]. However, this activity does not seem essential for the anti-apoptotic function of XIAP since an expression of XIAP mutant that has lost its ability to inhibit caspase-3 activity retained full ability to block ultra-violet-induced apoptosis [111].XIAP BIR2 IBM-binding groove binds the IBM of the large sub-unit of tetramer active effector caspases, and the linker region upstream of BIR 2 hinders the substrate binding pocket of caspase-3 and -7, preventing substrate accessibility [59,111,112,113,114]. Although the E3-ubiquitin ligase activity of XIAP seems dispensable, the analysis of cells from XIAP∆RING transgenic mice revealed the influence of the RING-dependent post-traductional modifications on XIAP-mediated caspase-3 inhibition [115]. Expression of the RING-deletion XIAP mutant did not compensate the deficiency of XIAP and increased caspase-3 activity and apoptosis in stem cells and thymocytes [115]. XIAP is able to induce the K48 ubiquitination of active caspases leading to their degradation [116], and the neddylation of caspase-7 inhibiting its activity [22].

cIAPs can interact with caspase-3 and caspase-7 in an IBM-dependent manner [59]. Moreover, cIAP1 seems able to interact with the pro-domain of caspases, independently of IBM [117]. Although unable to inhibit enzymatic activity of caspases [114], cIAPs can regulate the stability of active tetrameric caspases through a UPS (Ub Proteasome system)-dependent mechanism [59,117]. cIAP2 is able, at least *in vitro*, to induce a non degradative mono-ubiquitination of caspase-3 and -7 [118], suggesting a UPS-independent, Ub-dependent mechanisms of regulation.

### 5.5. Regulation of RIP1-Containing Caspase-Activating Platform Assembly

The serine/threonine kinases from RIP family are mediators in adaptive response to cellular stress caused by pathogen infections, inflammation or genotoxic stress (reviewed in [119]. They are important determinants of the response of cells, able to transduce survival or differentiation signals and to activate cell death pathways. RIP1 is a component of TNF-signalling pathway. It owns the homotypic interacting module death domain (DD) found in the death receptor Fas, TRAIL-R1 and TRAIL-R2, in the TNF signaling pathway adaptor TRADD, and in FADD and RAIDD, adaptors in caspase-8 and caspase-2 activating platforms, respectively. RIP1 is recruited via TRADD to TNFR1, and then is subjected to post-translational modifications including ubiquitination which determines its molecular function. When conjugated to K63-linked ubiquitin chains, RIP1 serves as a recognition signal for the recruitment of signaling complex leading to downstream activation of MAPK and NF-κB [120,121,122,123]. When ubiquitination is reduced, RIP1, through its kinase activity, promotes the assembly of the caspase-activating platform such as complex II that leads to caspase activation and cell death [121,123]. Such cytoplasmic RIP1-containing caspase-activating platform can also be formed independently of death receptor and is named RIPoptosome [94,95]. RIP1-containing complex can elicit apoptotic response, or caspase-independent cell death referred to as necroptosis, depending on the presence of RIP3, cellular FLICE-inhibitory protein (cFLIP) and the generation of reactive oxygen species [124].

**Figure 4:**
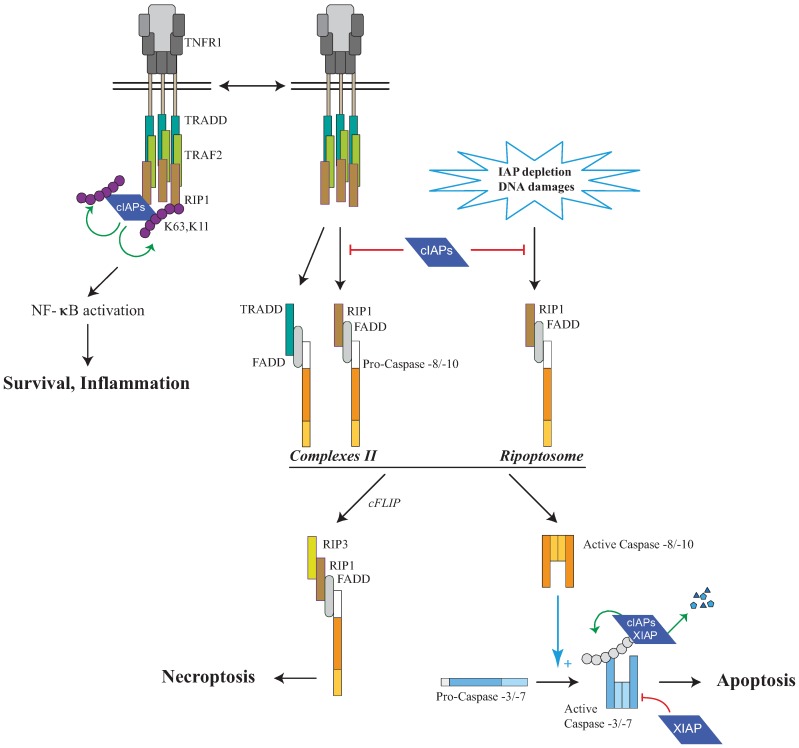
Regulation of RIP1-containing platforms by IAPs. Tumor Necrosis Factor Receptor 1 (TNFR1) stimulation induces the recruitment, to the receptor, of cIAPs and Receptor interacting protein 1 (RIP1) via the adaptors TNFR1-associated Death domain (TRADD) and TNFR associated factor (TRAF2). cIAPs trigger K63 self-ubiquitination and K11 and K63-ubiquitination of RIP1 leading to NF-κB activation and survival. In the absence of cIAPs, a secondary RIP1-containing cytoplasmic complex is formed (complex II) leading to cell death. A cytoplasmic RIP-1-containing complex named RIPoptosome can also be assembled, in the absence of Death Receptor stimulation, after DNA-damage-mediated IAP depletion or the use of synthetic IAP antagonists known to induce IAP degradation. RIP1-containing platform can lead to either caspase-8 or -10 activation and apoptosis, or caspase-independent cell death referred to as Necroptosis. Initiator caspases-8 or -10 induce the activating proteolytic processing of effector caspase-3 or -7 responsible for apoptosis. IAPs can control cell death at different levels: (1) cIAPs can induce the K63 and K11 ubiquitination of RIP1 allowing NF-κB activation and preventing the formation of complex II or RIPoptosome; (2) cIAPs and XIAP can induce K48-ubiquitination of RIP1 leading to its proteasomal degradation. (3) XIAP can directly inhibit the activity of processed effector caspase-3 or -7; (4) cIAPs and XIAP can induce ubiquitination and proteasome-mediated degradation of processed forms of caspase-3 or -7.

The recent analysis of *ciap1/xiap* and *ciap1/ciap2* double knockout mice demonstrated the importance of IAPs in the regulation of RIP1-dependent cell death. Indeed, the deletion of *ciap1* plus *ciap2* or *xiap* leads to the embryonic lethality, which is rescued or delayed by hemizygosity for *rip1* [88].RIP proteins are ubiquitination targets of cIAPs which can mediate the conjugation of K11, K48 and K63 and linear Ub chains [94,121,122,125,126,127,128]. Upon TNFR1 stimulation, cIAP1 induced self-ubiqitination and K11 and K63 poly-ubiquitination of RIP-1 required for the activation of the NF-κB signaling pathway [121,122,127,129]. In the absence of cIAPs, RIP1, in a non-ubiquitinated form, is unable to transduce survival pathway, and is recruited to a secondary cytoplasmic cell platform (complex-II) resulting to cell death (Figure 4). cIAP1 also prevents the assembly of secondary RIP1-containing cell death complexes after TRAILR, or CD95 stimulation [126]. Independently of the stimulation of TNFR members, the Ripoptosome formed in response to genotoxic stress, Tweak engagement or Toll-like receptor 3 stimulation is also negatively regulated by cIAP1, cIAP2 and XIAP [94,95] (Figure 4). cIAP1 could promote proteasomal-mediated degradation of component of RIPoptosome [94]. Ubiquitination of RIP1 could also inhibit the RIP1 kinase activity required for cell death complex assembly.

### 5.6. Mammalian Endogenous IAP Antagonists

Mammalian cells express large number of proteins that bear a potential IBM motif but their ability to bind and regulate IAPs and apoptosis remains to be investigated [23,130]. Most of them are expressed as a precursor into the mitochondrial. The best characterized are Smac/Diablo and HtrA2 [91,92]. They are released into the cytosol during apoptosis, after matured processing that removes the *N-*terminal mitochondrial import signal and then exposed the IBM to the *N-*extremity of the protein. Once cytosolic, they bind the IBM groove of IAPs, preventing them from binding caspases. Smac/Diablo can bind to both BIR3 and BIR2 domains and antagonizes XIAP inhibition of caspase-9 and caspase-3 [131,132]. Smac/Diablo can also interact with cIAP1 and cIAP2. It is generally admitted that Smac/Diablo could abrogate XIAP-mediated caspase inhibition while cIAPs could prevent Smac/Diablo from neutralizing XIAP (Figure 3). IAPs might also mediate the ubiquitination and degradation of these proteins [133,134]. Conversely, Smac/Diablo can promote auto-ubiquination and degradation of cIAPs [28,135]. Omi/HtrA2 is a serine protease that binds and inactivates cIAPs and XIAP by an irreversible proteolytic cleavage [136,137,138]. The transcriptional expression of Omi/HtrA2 is controlled by p53 and then up-regulated after DNA damages [138]. Another mitochondrial protein translocated into the cytosol in response to apoptotic stimuli and that can antagonize IAP is the septin-like protein ARTS [139]. Although it does not harbour conventional IBM, it efficiently binds XIAP and induces its ubiquitin/proteasomal-dependent downregulation [140,141]. Inactivation of ARTS in mice leads to an increased incidence of leukemia/lymphoma and hematopoietic stem cells and progenitors are significantly more resistant to apoptotic stimuli. This phenotype is partly reversed by the inactivation of XIAP [142], demonstrating the importance of ARTS-mediated XIAP regulation in hematopoietic compartment. 

As Jafrac2 in drosophila [72], GSPT1/eRF3 (G1 to S phase transition protein/eukaryotic Release Factor 3) is an IBM-containing ER protein. The IBM motif is exposed after ER-stress that induces the removal of the *N-*terminal endoplasmic reticulum signal peptide. GSPT1/eRF3-cIAPs interaction selectively stimulates cIAP1 auto-ubiquitination and degradation [143].

In contrast to drosophila, mammalian IAP antagonists seem dispensable for apoptosis induction. In most cases, deletion or neutralisation of IAPs did not result in apoptosis, except in some tumour cells in which depletion of IAPs triggered a spontaneous assembly of Ripoptosome [94,95] or resulted to the production of TNFα, which triggers cell death via an autocrine pathway [121,144,145,146,147,148]. Apoptotic stimuli such as glucocorticoid or DNA damaging agents can induce the auto-ubiquitination and degradation of cIAPs [149] which could result to RIPoptosome assembly [94] but the role of IAP antagonists in these processes was not determined. The physiological functions of IBM-bearing proteins are not established. The role and the importance of most of these molecules in the regulation of IAPs is not much documented and more investigations will be required for determining their level of action in the regulation of apoptosis or in the regulation of other functions of IAPs such as innate immunity in mammals. One other possibility is that IAPs, through their E3-ubiquitin ligase activity could catalyse the destruction of IBM-containing proteins. 

## 6. Conclusion

Although IAPs were first described as apoptotic regulators, the importance of this function in mammals has long been discussed. However, the analysis of cells derived from IAPs or IAP antagonist-deleted mice revealed the importance of IAPs in adaptive response of cells to specific cellular or environmental injuries [4]. For example, XIAP and NAIP have been involved in the adaptive response of neuronal cells to hypoxic-ischemia injury [150,151,152]. Furthermore, abnormalities in IAP expression have been observed in diseases linked to a deregulation of cell death pathways. A reduced expression of XIAP has been found in neurodegerative disorders such as Huntington’s and Wilson’s disease [153,154]. Inversely, overexpression of IAPs has been detected in number of tumour samples and correlated with bad prognosis or poor response to chemotherapy [155]. The importance of IAPs in adaptive response to cellular stress was strengthened by the discovery of the presence of IAP-translational and transcriptional regulation-mechanisms which keep high the level of expression of IAPs under stressful conditions such as hypoxia, anoxia, serum deprivation, reticular or genotoxic stress [156,157,158,159]. However, the mechanisms regulating IAP activity remain poorly understood. A number of mammalian IBM-bearing proteins, potential IAP regulators remain to be characterised and post-translational regulations such as phosphorylation, oligomerization and subcellular localization are poorly documented.

The knowledge of the mechanisms of interaction of XIAP with caspases and IAP antagonists provided very potent tools for the design of synthetic IAP antagonists named Smac mimetics (SMs) aiming to inhibit the anti-apoptotic function of IAPs. These molecules are currently under clinical evaluation and give promising results in treating cancer, in association with conventional therapy or death receptor agonists (for review, see [160]). They were also helpful tools for the investigation of IAP functions. SMs appeared to be potent inhibitors of cIAPs, mediating their auto-ubiquitination and rapid proteasomal-mediated degradation [144,145,161]. SMs considerably alter the NF-κB activating signalling pathway, stimulating the production of pro-inflammatory cytokines including TNF-α [121,144,145,146]. Thus, SMs highlighted the important role of cIAPs in the regulation of NF-κB activation and innate immunity. The consequences of SMs on immune system *in vivo*, and the use of cIAPs as potential therapeutic targets for inflammatory or immune disorders are still important questions that need to be addressed.
